# Nano-encapsulated Yucca extract as feed additives: Ruminal greenhouse gas emissions of three forages

**DOI:** 10.1186/s13568-024-01803-3

**Published:** 2024-12-24

**Authors:** Edwin Oswaldo Botia-Carreño, Mona M. M. Y. Elghandour, Ameer Khusro, Desiderio Rodriguez Velazquez, Susanne Kreuzer-Redmer, Abdelfattah Z. M. Salem

**Affiliations:** 1 Facultad de Medicina Veterinaria y Zootecnia, Universidad Nacional Autónoma de México (UNAM), Av. Universidad 3000, 04510 Coyoacán, Ciudad de Mexico Mexico; 2 Facultad de Medicina Veterinaria y Zootecnia, Universidad Autónoma del Estado de México, El Cerrillo Piedras Blancas, 50295 Toluca, Estado de México Mexico; 3 Department of Research Analytics, Saveetha Dental College and Hospitals, Saveetha Institute of Medical and Technical Sciences, Saveetha University, Chennai, 600077 India; 4 Centre for Animal Nutrition and Animal Welfare Sciences, Clinical Department for Farm Animals and Food System Safety, University of Veterinary Medicine Vienna, Veterinärplatz 1, 1210 Vienna, Austria

**Keywords:** Chitosan, Nanoparticles, Greenhouse gases, Forages, Rumen fermentation, *Y. schidigera*

## Abstract

Reducing greenhouse gas (GHG) emissions from livestock is a crucial step towards mitigating the impact of climate change and improving environmental sustainability in agriculture. This study aimed to evaluate the effects of *Yucca schidigera* extract, chitosan, and chitosan nanoparticles as feed additives on in vitro GHG emissions and fermentation profiles in ruminal fluid from bulls. Total gas, CH_4_, CO, and H_2_S emissions (up to 48 h), rumen fermentation profiles, and CH_4_ conversion efficiency were measured using standard protocols. The experiments involved supplementing 0.25, 0.5, and 1 mL/g dry matter (DM) of additives in different forages (alfalfa hay, corn silage, and oats hay). The chemical composition of forage showed suitable levels of DM, ash, crude protein, acid detergent fiber, neutral detergent fiber, lignin, and metabolizable energy. The addition of these supplements increased asymptotic gas production across all forages while simultaneously reducing CH_4_, CO, and H_2_S emissions, though the extent of reduction varied depending on forage type. Moreover, the treatments improved fermentation profiles, including pH and dry matter digestibility, and significantly influenced CH_4_ conversion efficiency (CH_4_:ME, CH_4_:OM, and CH_4_:SCFA; *P* < 0.05). These results underscore the potential of *Y. schidigera* extract, chitosan, and chitosan nanoparticles as effective strategies for mitigating GHG emissions from ruminants given these promising in vitro findings. Further in vivo studies are recommended to validate their efficacy under real-world conditions, which could pave the way for practical applications in the field.

## Key points

1. *Y. schidigera* extract, chitosan, and chitosan nanoparticles significantly increased total gas production in *in vitro* studies with rumen fluid inoculum from bulls.
2. Despite the increase in total gas production, these additives effectively reduced CH_4_, CO, and H_2_S emissions, with reductions varying by forage type.
3. Additives improved ruminal fermentation parameters, such as pH and dry matter digestibility, and influenced CH_4_ conversion efficiency.
4. *Y. schidigera* extract, chitosan, and chitosan nanoparticles demonstrate strong potential as ruminal modifiers for mitigating GHG emissions, warranting further in vivo testing.

## Introduction

Livestock industries are responsible for releasing approximately 5% of total greenhouse gases (GHG) emitted worldwide (Lileikis et al. 2023). The uncontrolled growing population of the world has increased the demand for livestock products, which ultimately led to the enhancement of GHG emissions from animals. The emission of GHG from ruminants and non-ruminants is one of the contributing factors towards global warming (Khusro et al. 2022). Among diversified livestock, ruminants have a unique digestive system with diverse groups of microflora that convert fibrous plant constituents of the diet into high-value products via an enteric fermentation process, thereby giving rise to the emission of GHG which contributes efficiently to the global warming effect (Lileikis et al. 2023; Elghandour et al. 2023).

Microbes present in the rumen use Embden-Meyerhoff and pentose phosphate pathways for fermenting hexose and pentose to pyruvate. The pyruvate produced is then converted into varied end products viz. formate, acetate, propionate, butyrate, lactate, succinate, carbon dioxide (CO_2_), and hydrogen (H_2_). Further, methanogens use H_2_, CO_2_ or formate as the main substrate to emit methane (CH_4_) (Santoso et al. 2004). Ruminants produce about 95% of CH_4_ via fermentation of forage, while approximately 99% are exhaled through the nose and mouth, which causes a significant loss of energy as per the diet consumed (Soliman 2022). Therefore, mitigating GHG emissions from ruminants has become essential due to its adverse impact on the environment and the climate. Over the past few years, significant efforts have been undertaken to mitigate GHG emissions from ruminants. In general, the inhibition of methanogenesis, reducing H_2_ production during fermentation, and utilization of H_2_ via alternative pathways are the main principles to achieve GHG mitigation from ruminants (Greening et al. 2019). Currently, dietary manipulation is considered a pronounced strategy to achieve this desired outcome (Khusro et al. 2022). Plants are being used as an alternative to chemical feed additives and antibiotics which modulate the ruminal fermentation process, improve nutrient digestibility, and mitigate GHG emissions (Zeid et al. 2019). However, the GHG reduction potency of these natural feed supplements depends on the source, type, geographical location, and presence of diverse bioactive components in it (Patra 2012).

*Yucca schidigera* (Agavaceae), a desert flowering plant, is native to Baja California, Mojave, Sonoran, Great Basin, in the southwestern USA and northwestern Mexico. It is also called “Spanish Dagger” or “Yucca” (Xu et al. 2010). *Y. schidigera* extract is an ample source of steroidal saponins with diverse biological activities (Johnson et al. 2023). Previous studies reported CH_4_ mitigation ability of *Y. schidigera* extract in vitro (Pen et al. 2006) and in vivo (Wang et al. 2009), indicating its efficacy as a potent feed additive for ruminants in the future.

In recent years, chitosan (a linear polysaccharide) has been extensively used for the fabrication of nanoparticles (El-Naggar et al. 2022; Priyamvadan et al. 2024). Chitosan nanoparticles are cationic polymeric nanoparticles that show interesting surface properties. These nanoparticles are not only biocompatible, biodegradable, safe, and non-toxic in nature but also easy to synthesize and exhibit disparate industrial applications (Yin et al. 2017; Kugarajah et al. 2023; Revathi et al. 2024). However, physical and chemical methods-based synthesized chitosan nanoparticles reveal several drawbacks viz. utilization of high pressure, temperature, and different toxicants. Thus, green synthesis of chitosan nanoparticles is considered a pivotal alternative to the physical and chemical methods (El-Naggar et al. 2022).

Current reports showed the unique potentiality of chitosan in the modulation of rumen fermentation and nutrient digestibility in livestock. In addition to this, chitosan shows promising impact on feed intake, digestion, rumen microflora, and biogas production ability of ruminants (Jiménez-Ocampo et al. 2019). Surprisingly, the applications of plant-based synthesized chitosan nanoparticles as feed additives are subtle in livestock sciences. Thus, this investigation hypothesizes that the inclusion of *Y. schidigera* extract, chitosan, and *Y. schidigera* extract-based chitosan nanoparticles into animal’s diet significantly affects GHG production from livestock. Considering this, the present study was conducted to decipher the efficacy of *Y. schidigera* extract, chitosan, and *Y. schidigera* extract-based chitosan nanoparticles at varied concentrations in the mitigation of GHG from bulls.

## Materials and methods

### Chemical analysis of forages used

Alfalfa hay (*Medicago sativa*), corn silage (*Zea mays*), and oats hay (*Avena sativa*) were collected from Toluca region, State of Mexico, Mexico, and dehydrated in a forced air oven at 64 °C for 72 h. A proximate chemical composition analysis of the dried forage samples was performed following the methodology described in AOAC (AOAC 1997). For the measurement of the fiber fraction, an ANKOM200 Fiber Analyzer Unit (ANKOM Technology Corp., Macedon, NY) was used (AOAC 1997). The neutral detergent fiber (NDF) and acid detergent fiber (ADF) fractions were determined using the methodology described by Van Soest et al. (1991).

### Synthesis of chitosan nanoparticles

The aqueous extract of *Y. schidigera* (Biolíquid 3000®) was donated by the company Baja Agro International S.A. de C.V. AGROIN® (Ensenada, Baja California, northwest of Mexico). *Y. schidigera* extract contains steroidal saponins. Additionally, the presence of resveratrol and some new phenolic compounds with a very unusual spiral structure, named yuccaols A–E and yuccaone A, has been found.

The encapsulation process of *Y. schidigera* extract was carried out in two stages using 100 mL of a 1% acetic acid solution. In the first stage, 0.5 g of Pluronic F127® (Sigma-Aldrich®, Toluca, Mexico) was weighed and slowly added to 50 mL of the 1% acetic acid solution under constant agitation at 600 rpm until completely dissolved, and then 0.3 g of chitosan (Sigma-Aldrich®, Toluca, Mexico; purity > 90%) was added to it.

In the second stage, 0.1 g of sodium tripolyphosphate (Sigma-Aldrich®, Toluca, Mexico) was weighed and added to the remaining 50 mL of 1% acetic acid solution until completely dissolved. Then 0.18 mg of *Y. schidigera* extract was gradually mixed into it. After that, the mixture from the second stage was gradually added to the mixture from the first stage and maintained under constant mechanical agitation at 600 rpm until a complete mixture was obtained (Piacente et al. 2005). Evaluations were conducted with different amounts of *Y. schidigera* extract to find a stable mixture that would interact appropriately with the chitosan solution, followed by macroscopic observations up to 72 h to assess possible changes in the phases of nano-emulsion.

### Determination of particle size and polydispersity index (PDI)

Particle size was measured at 25 °C using a Malvern laser particle size analyzer (Zetasizer Ver. 7.11, UK) and PDI was determined (Ribeiro et al. 2014).

### In vitro incubation

**Fig. 1:**
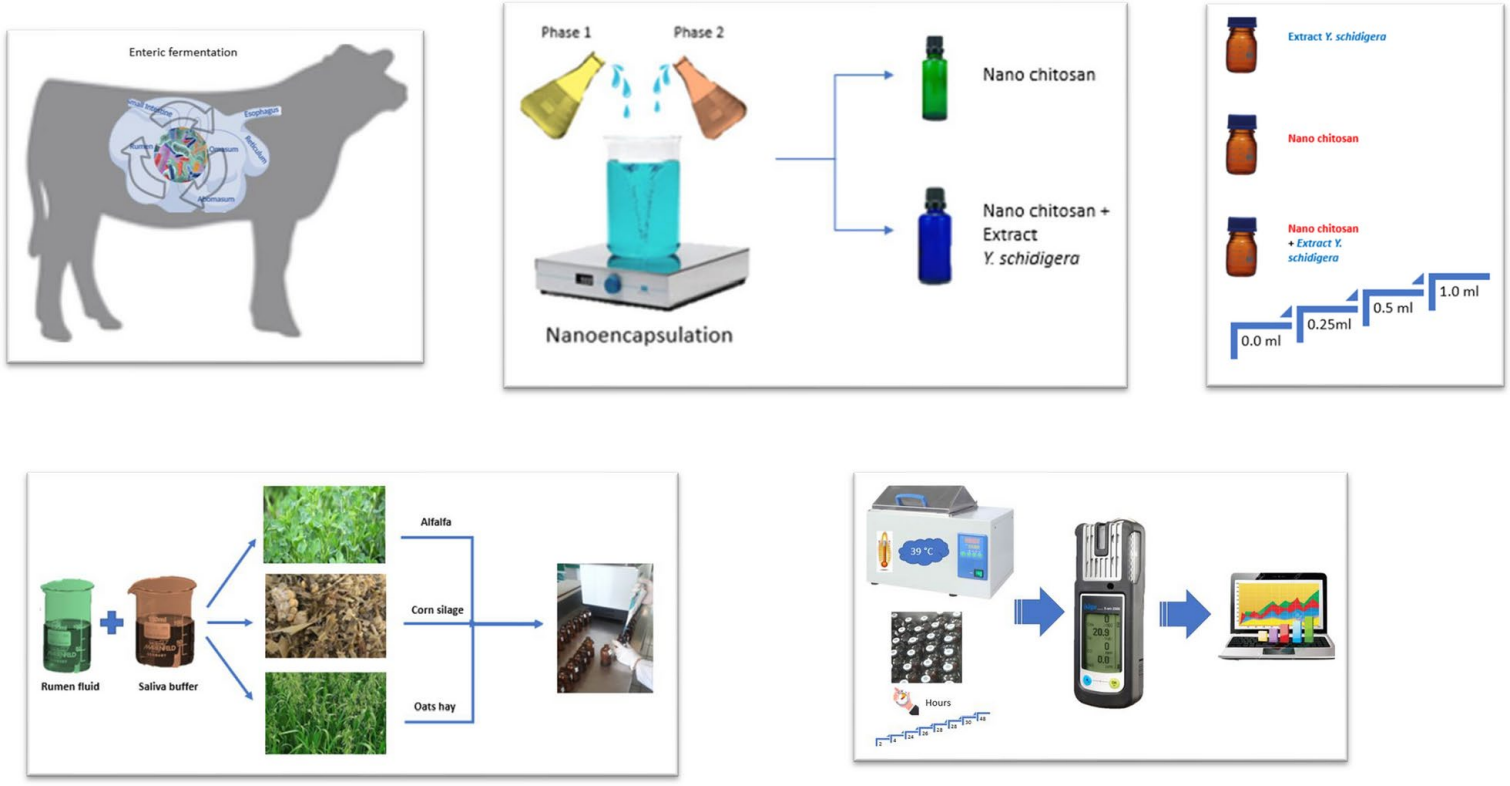
Methodology flowchart that summarizes the experimental design providing a visual overview of the research conducted.

Alfalfa hay, corn silage, and oats hay were ground using a hammer mill (Thomas Wiley® Laboratory Mill model 4, Thomas Scientific™, Swedesboro, NJ, USA), as described by Alvarado-Ramírez et al (2024) for achieving a particle size of ≤ 1 mm to ensure homogeneity. For in vitro incubation, a total of 435 vials were incubated [(3 vials of each triplicated sample within each of the 3 forages (alfalfa hay, corn silage, and oat hay)] with 4 different types of additives [negative control (without extract), positive control (chitosan), crude extract of *Y. schidigera*, and chitosan nanoparticles)] at different doses (0, 0.25, 0.5, and 1.0 mL/g DM) in 3 runs on different weeks with 3 vials as blank (i.e., rumen fluid only) for 48 h. The animals, from which the rumen fluid was taken, received a diet composed of hay and Purina® concentrated feed (Toluca, State of Mexico, Mexico) in a 50:50 ratio, with free access to fresh water before slaughter. The animals, crossbreeds of Brahman with Limousin and Brahman with Charolais, were slaughtered at the municipal slaughterhouse in Toluca, State of Mexico, Mexico, in accordance with the Official Mexican Standard NOM-033-SAG/ZOO-2014. The ruminal contents of each animal were taken directly at the slaughter line. Samples of ruminal contents (filtered through eight layers of gauze) were collected in thermos flasks (previously filled with distilled water at 39 °C to avoid thermal shock to the rumen fluid), insufflating CO_2_ into the headspace to ensure the environment remained anaerobic, and transported to the laboratory within 30 min. After transport, the top layer of ruminal contents was discarded, and the remaining portion was mixed and blended under a CO_2_ headspace for 1 min to remove any additional particles and/or attached organisms. The combined fluid and contents were strained through 6 layers of cheesecloth to form the inoculum for in vitro fermentation, —Fig. 1.

### Ruminal in vitro total gas, CH_4_, CO, and H_2_S gas production

For each of the treatments, three replicates were performed in each incubation cycle, three blank negative controls (without substrate) per inoculum, as well as the positive control of chitosan (same extract doses), to correct the readings and reduce the presence of errors in the data. The vials were incubated in a water bath at a constant temperature of 39 °C for 48 h, during which records were noted at 2, 4, 24, 26, 28, 30, and 48 h after inoculation using the methodology described by Alvarado-Ramírez et al. (2023). Total gas production was measured in psi, and CH_4_, CO, and H_2_S productions were also measured using a diffusion gas detector (Dräger Safety X-am 20500 MONITOR, Lübeck, Germany). After each measurement, the gas from each bottle was dispersed to avoid accumulation and maintain a constant gas pressure of 48 kPa.

### Ruminal pH and dry matter degradability (DMD)

After 48 h, the content of each vial was filtered through F57 filter bags (ANKOM Technology Corp., Macedonia, NY, USA) with a porosity of 25 μm, separating the solid fraction from the liquid fraction in beakers. In the liquid fraction, pH was measured using a potentiometer (HALO^®^ wireless pH electrode model HI11102, Hanna^®^ Instruments, Woonsocket, RI, USA). These bags containing the solid fraction were washed using tap water and dried at 60 °C for 48 h to obtain the dry weight result. This value was used for calculating DMD, as described by Elghandour et al. (2023).

### Calculations and statistical analyses

For calculating asymptotic gas production, production rate, and lag phase time of the measured gases, the production volumes (mL/g DM incubated) of total gas (psi), CH_4_, CO, and H_2_S were used, as per NLIN procedure of the Statistical Analysis System (SAS 2002) and the model proposed by France et al. (2000). Metabolizable energy (ME; MJ/kg DM) was calculated using the equation proposed by Menke et al. (1979), while the short-chain fatty acids (SCFA; mmol 200 per mg DM) were calculated according to the methodology of Getachew et al. (2002). Additionally, the efficiency of CH_4_ conversion was evaluated through the production of CH_4_ per unit of SCFA (CH_4_:SCFA), ME (CH_4_:ME), and organic matter or OM (CH_4_:OM) in mmol/mmol, g/MJ, and mL/g, respectively.

For in vitro incubation experiments, a completely randomized experimental design with a factorial arrangement (3 × 4 × 4) was used, where the first factor was the forages used (alfalfa hay, corn silage, and oats hay), the second factor was the types of additives used [(negative control i.e. without extract), positive control (chitosan), and chitosan nanoparticles], and the third factor was the doses (0.25, 0.5, and 1 mL/g DM) of each type of additives used. The data from the three replications of each treatment in each run were considered as mean, and these values were used as the experimental unit for each treatment. For the data analysis, the GLM procedure of SAS (SAS 2002) and the statistical model listed below was used:

$$ \begin{aligned} {\text{Y}}_{{{\text{ijk}}}} & = {\upmu } + {\upalpha }\;{\text{CH}}_{{\text{i}}} + {\text{TE}}_{{\text{j}}} + {\text{EX}}_{{\text{k}}} + \left( {{\text{CH}} \times {\text{TE}}} \right)_{{{\text{ij}}}} + \left( {{\text{CH}} \times {\text{EX}}} \right)_{{{\text{ik}}}} \\ & \quad + \left( {{\text{TE}} \times {\text{EX}}} \right)_{{{\text{jk}}}} + \left( {{\text{CH}} \times {\text{TE}} \times {\text{EX}}} \right)_{{{\text{ijk}}}} + {\upvarepsilon }_{{{\text{ijk}}}} \\ \end{aligned} $$

where Y_ijk_ is the response variable, μ is the overall mean, CH_i_ is the effect of the type of forage, TE_j_ is the effect of the type of extract, EX_k_ is the effect of the additive doses, (CH × TE)_ij_ is the effect of the interaction between the type of forage and the type of additive, (CH × EX)_ik_ is the effect of the interaction between the type of forage and the additive doses, (TE × EX)_jk_ is the effect of the interaction between the type of additive and the additive doses, (CH × TE × EX)_ijk_ is the effect of the interaction between the type of forage and type of additive and doses of additives, and ε_ijk_ is the experimental error. The comparison of means was performed using Tukey's test and were considered significantly different when *P* ≤ 0.05. The linear and quadratic effects of the type of forage as well as the extract doses were calculated.

## Results

### Chemical composition of forages

The chemical composition of alfalfa hay, corn silage, and oats hay is displayed in Table 1. Results showed higher DM, ash, crude protein (CP), ADF, and lignin contents of alfalfa hay and oats hay than that of corn silage. The ME (2.45 MJ/kg DM) and ether extract (3.2%) values were comparatively higher for corn silage with respect to alfalfa hay and corn silage. On the other hand, oats hay showed higher NDF (58%) than alfalfa hay (41.6%) and corn silage (45%).

**Table 1:** Chemical composition of forages used as substrates

Forage	DM (%)	Ash (%)	CP (%)	Ether Extract (%)	NDF (%)	ADF (%)	Lignin (%)	ME (MJ/kg DM)
Alfalfa hay	90.3	11	19.2	2.5	41.6	32.8	7.6	1.87
Corn silage	35.1	4.3	8.8	3.2	45	28.1	2.6	2.45
Oats hay	91.9	8.5	9.1	2.2	58	36.4	6.5	2.27

DM, dry matter; CP, crude protein; NDF, neutral detergent fiber; ADF, acid detergent fiber; ME, metabolizable energy

### Particle size and PDI of chitosan nanoparticles

The mean diameter and PDI of synthesized chitosan nanoparticles were observed as 244.8 nm and 0.212, respectively (Table 2).

**Table 2:** Characterization of chitosan nanoparticles in terms of size and PDI

Size (nm)	St. Dev	PDI
244.8	15.85	0.212

St. Dev, standard deviation; PDI, polydispersity index

###  Gas production kinetics and production

The effect of different additives at varied doses (0.25, 0.5, and 1 mL/g DM) on ruminal gas production kinetics and total gas production from bulls using alfalfa hay, corn silage, and oats hay as forages is shown in Table 3. Using alfalfa hay as forage, the additives (chitosan, extract, and chitosan nanoparticles) at varied concentrations showed higher asymptotic gas production than the control (no additive). The interaction between additive types and additive doses showed a significant (*P* = 0.0093) increment in asymptotic gas production. Likewise, the rate of gas production in the presence of extract was estimated higher than that of control and other additives (chitosan and chitosan nanoparticles). The interaction between additive types and additive doses showed a significant (*P* = 0.0147) effect on the rate of gas production. The inclusion of extract at different concentrations reduced the lag period as compared to the control and additives. Total gas production was increased significantly (*P* < 0.05) from 6 to 48 h in the presence of all additives. Moreover, the supplementation of extract (1 mL/g DM) exhibited maximum total gas production of 484.9 mL gas/g DM incubated with respect to the control (240.1 mL gas/g DM incubated) and other additives [chitosan (311.5 mL gas/g DM incubated) and chitosan nanoparticles (250.5 mL gas/g DM incubated)].

**Table 3:** Effect of different additives at varied doses (0.25, 0.5, and 1 mL/g dietary DM) on ruminal gas production kinetics and total gas production from bulls using alfalfa hay, corn silage, and oats hay as forages

Forages (FOR)	Types of additives (TA)	Additive doses (AD; mL/g DM)	Gas production kinetics^1^			Gas production (mL gas/g DM incubated)		
			*b*	*c*	*Lag*	6 h	24 h	48 h
Alfalfa hay	No additive	0	240.1	0.274	1.663	134.0	154.9	240.1
	Chitosan	0.25	276.4	0.348	1.260	49.9	98.6	276.3
		0.5	310.9	0.213	1.039	53.7	108.1	311.1
		1	310.8	0.210	1.032	52.7	109.5	311.5
	Crude extract	0.25	341.8	0.356	0.627	72.7	174.7	340.4
		0.5	408.0	0.329	0.547	102.5	210.1	407.7
		1	485.2	0.338	0.545	117.3	242.4	484.9
	Chitosan nanoparticles	0.25	246.0	0.168	1.875	144.2	163.1	246.9
		0.5	248.9	0.163	1.385	135.2	151.6	248.8
		1	250.7	0.171	2.045	143.3	162.0	250.5
	TA		< 0.0001	< 0.0001	< 0.0001	< 0.0001	< 0.0001	< 0.0001
	AD		0.0022	0.0068	0.1734	0.0007	0.0041	0.002
	TA × AD		0.0093	0.0147	0.3235	< 0.0001	0.0048	0.0096
Corn silage	No additive	0	262.9	0.150	0.838	155.9	172.5	262.9
	Chitosan	0.25	349.2	0.283	1.099	60.4	113.9	350.3
		0.5	378.3	0.498	1.036	71.0	123.1	379.0
		1	344.2	0.589	1.137	68.5	112.8	344.3
	Crude extract	0.25	467.6	0.365	0.597	129.6	235.2	467.0
		0.5	481.7	0.314	0.634	138.9	243.7	481.4
		1	414.7	0.276	0.847	144.3	231.2	414.2
	Chitosan nanoparticles	0.25	262.7	0.159	1.184	150.9	168.1	262.9
		0.5	268.6	0.151	0.663	161.3	178.0	268.2
		1	263.9	0.153	0.981	153.0	170.1	262.1
	TA		< 0.0001	< 0.0001	0.0041	< 0.0001	< 0.0001	< 0.0001
	AD		0.2028	0.8545	0.7665	< 0.0001	0.0011	0.2202
	TA × AD		0.6995	0.8682	0.3843	< 0.0001	0.0002	0.6887
Oats hay	No additive	0	260.6	0.145	1.048	133.3	152.4	260.3
	Chitosan	0.25	360.8	0.816	1.071	38.8	98.4	361.0
		0.5	393.6	0.802	1.053	44.6	112.6	394.1
		1	378.0	0.997	1.039	45.8	110.8	378.2
	Crude extract	0.25	368.3	0.331	0.806	116.9	216.1	368.8
		0.5	383.0	0.364	0.837	65.4	166.4	382.7
		1	424.2	0.313	0.672	129.6	227.2	423.7
	Chitosan nanoparticles	0.25	267.9	0.153	0.984	137.7	157.6	268.7
		0.5	275.4	0.146	1.173	139.0	160.1	274.5
		1	288.9	0.147	1.356	150.4	170.8	287.8
	TA		< 0.0001	0.0153	0.0003	< 0.0001	< 0.0001	< 0.0001
	AD		0.1082	0.7463	0.0353	0.0663	0.2797	0.107
	TA × AD		0.5357	0.5068	0.0699	0.7203	0.9974	0.5326
SEM pooled^2^			13.60	0.0474	0.0931	3.72	6.07	13.53
*P* value:								
FOR			0.0005	0.0162	< 0.0001	< 0.0001	< 0.0001	0.0005
TA			< 0.0001	< 0.0001	< 0.0001	< 0.0001	< 0.0001	< 0.0001
AD			0.0217	0.8416	0.0669	< 0.0001	0.0037	0.0223
FOR × TA			0.0002	< 0.0001	< 0.0001	< 0.0001	0.0504	0.0003
FOR × AD			0.0083	0.8196	0.3013	< 0.0001	0.0011	0.0075
TA × AD			0.2242	0.6941	0.1287	< 0.0001	0.0129	0.2157
FOR × TA × AD			0.0774	0.7479	0.3101	< 0.0001	0.0003	0.0774

^1^*b* = asymptotic total gas production (mL/g DM); *c* = rate of total gas production (mL/h); *Lag* = initial delay before total gas production begins (h)

^2^SEM = standard error of mean

FOR, Forages; TA, Types of additives; AD, Additive doses

Using corn silage as forage, the additives showed higher asymptotic total gas production (*P* < 0.05) than the control. However, asymptotic gas production was observed maximum in the presence of extract, which was estimated as 467.6, 481.7, and 414.7 mL/g DM at 0.25, 0.5, and 1 mL/g DM, respectively. Similarly, the rate of gas production increased due to the supplementation of all additives as compared to the control. Chitosan at 0.5 and 1 mL/g DM doses showed maximum rate of gas production of 0.498 and 0.589 mL/h, respectively. On the other hand, the supplementation of extract at 0.25 and 0.5 mL/g DM reduced the lag period from 0.838 h (control) to 0.597 and 0.634 h. The inclusion of chitosan (344.3 mL gas/g DM incubated) and extract (481.4 mL gas/g DM incubated) exhibited total gas production higher than the control (262.9 mL gas/g DM incubated) at 48 h. Surprisingly, the supplementation of varied concentrations of chitosan nanoparticles revealed total gas production more or less similar to the control.

The supplementation of additives in oats hay enhanced asymptotic gas production significantly (*P* < 0.0001) with respect to the control. Maximum asymptotic total gas production of 424.2 mL/g DM was obtained by supplementing 1 mL/g DM of extract. Similarly, the rate of gas production in treated groups was estimated higher (*P* = 0.0153) than the control. The inclusion of chitosan at 1 mL/g DM enhanced the rate of gas production (0.997 mL/h) as compared to the control (0.145 mL/h). On the other hand, the supplementation of extract at varied concentrations showed a comparatively lower lag period (0.806 to 0.672 h; *P* = 0.0003) with respect to the control and other additives. The incorporation of all additives depicted total gas production higher (*P* < 0.0001) than the control (260.3 mL gas/g DM incubated) at 48 h.

###  CH_4_ gas production

The effect of different additives at varied doses on ruminal CH_4_ production from bulls using alfalfa hay, corn silage, and oats hay as forages is summarized in Table 4. In the presence of alfalfa hay as forage, the asymptotic CH_4_ production was reduced when the diet was supplemented with different doses of extract. The supplementation of extract (1 mL/g DM) also showed a lower rate of CH_4_ production than the control. All the additives increased the lag period with respect to the control, but the effect was not significant (*P* = 0.7802). On the other hand, the inclusion of chitosan (38.54 mL gas/g DM incubated and 12.38 mL CH_4_/100 mL gas) and extract into the diet mitigated CH_4_ production up to 48 h as compared to the control. The supplementation of chitosan nanoparticles enhanced CH_4_ production up to 48 h as compared to the control.

**Table 4:** Effect of different additives at varied doses (0.25, 0.5, and 1 mL/g dietary DM) on ruminal CH_4_ production from bulls using alfalfa hay, corn silage, and oats hay as forages

Forages (FOR)	Types of additives (TA)	Additive doses (AD; mL/g DM)	CH_4_ production kinetics^1^			CH_4_ production (mL gas/g DM incubated)			CH_4_ (mL CH_4_/100 mL gas)		
			*b*	*c*	*Lag*	6 h	24 h	48 h	6 h	24 h	48 h
Alfalfa hay	No additive	0	44.09	0.009	2.221	1.12	8.80	43.98	0.83	5.67	18.31
	Chitosan	0.25	50.20	0.012	2.385	0.26	5.63	50.14	0.53	5.67	18.18
		0.5	46.51	0.014	2.428	0.27	4.66	46.43	0.50	4.30	14.95
		1	38.60	0.014	2.427	0.25	3.61	38.54	0.47	3.30	12.38
	Crude extract	0.25	36.01	0.011	2.394	0.81	5.25	35.94	1.10	3.03	10.64
		0.5	43.26	0.010	2.330	0.73	7.31	43.12	0.70	3.47	10.60
		1	40.20	0.008	2.285	0.95	6.89	40.00	0.80	2.83	8.28
	Chitosan nanoparticles	0.25	43.65	0.009	2.245	1.45	7.39	43.65	1.01	4.57	17.88
		0.5	43.37	0.009	2.204	2.87	8.80	43.29	2.13	5.80	18.48
		1	45.70	0.007	2.996	5.02	9.41	45.57	3.51	5.79	18.20
	TA		0.1301	< 0.0001	0.7802	< 0.0001	0.0001	0.1246	< 0.0001	< 0.0001	< 0.0001
	AD		0.56	0.1778	0.4187	< 0.0001	0.5068	0.5538	0.0015	0.3702	0.0667
	TA × AD		0.1253	0.1147	0.3587	< 0.0001	0.171	0.1315	< 0.0001	0.0228	0.2463
Corn silage	No additive	0	50.11	0.010	2.308	2.28	10.05	50.08	1.44	5.78	18.91
	Chitosan	0.25	52.61	0.016	2.395	0.51	4.62	52.81	0.83	4.07	15.11
		0.5	63.90	0.013	2.384	0.69	6.80	63.89	0.97	5.53	16.88
		1	60.10	0.012	2.403	0.89	5.14	60.27	1.30	4.53	17.44
	Crude extract	0.25	39.03	0.009	2.366	0.96	5.21	38.85	0.73	2.20	8.27
		0.5	37.19	0.009	2.306	0.97	6.85	37.04	0.70	2.83	7.71
		1	36.83	0.008	2.251	1.06	7.58	36.79	0.73	3.27	8.94
	Chitosan nanoparticles	0.25	60.44	0.013	2.282	2.63	8.64	60.01	1.78	5.17	22.76
		0.5	43.73	0.007	2.229	4.16	8.17	44.20	2.59	4.59	16.51
		1	35.19	0.008	2.192	3.35	8.86	34.81	2.21	5.21	13.28
	TA		< 0.0001	0.0144	< 0.0001	< 0.0001	< 0.0001	< 0.0001	< 0.0001	< 0.0001	< 0.0001
	AD		0.4086	0.0982	0.0282	0.1807	0.0183	0.3961	0.2506	0.021	0.7109
	TA × AD		0.1842	0.0929	0.4129	0.4186	0.7931	0.1892	0.3652	0.3852	0.091
Oats hay	No additive	0	57.25	0.008	2.206	3.16	11.34	57.20	2.36	7.47	22.01
	Chitosan	0.25	67.20	0.015	2.446	0.17	4.53	67.40	0.43	4.63	18.62
		0.5	68.80	0.013	2.467	0.19	3.26	69.01	0.43	2.90	17.52
		1	77.84	0.012	2.428	0.25	6.06	77.91	0.53	5.47	20.63
	Crude extract	0.25	40.63	0.008	2.272	0.83	7.75	40.63	0.70	3.60	11.06
		0.5	59.78	0.015	2.428	0.37	5.06	59.82	0.57	3.00	15.65
		1	42.28	0.008	2.405	0.91	7.51	42.35	0.70	3.27	9.84
	Chitosan nanoparticles	0.25	49.42	0.009	2.110	4.33	12.48	49.13	3.13	7.91	18.30
		0.5	47.77	0.010	2.238	2.15	8.34	47.76	1.56	5.22	17.40
		1	49.54	0.010	2.172	3.61	11.48	49.36	2.39	6.72	17.20
	TA		0.0012	0.0414	0.0002	0.0003	0.0156	0.001	0.0028	0.0004	<.0001
	AD		0.4016	0.1637	0.1221	0.6217	0.449	0.3981	0.6727	0.504	0.3716
	TA × AD		0.0917	0.7972	0.5557	0.847	0.6492	0.0945	0.8794	0.465	0.0497
SEM pooled^2^			4.321	0.0012	0.053	0.360	0.883	4.318	0.264	0.481	1.257
*P* value:											
FOR			< 0.0001	0.9282	0.5773	0.1491	0.0865	< 0.0001	0.2603	0.0133	0.0045
TA			< 0.0001	< 0.0001	0.2164	< 0.0001	< 0.0001	< 0.0001	< 0.0001	< 0.0001	< 0.0001
AD			0.4436	0.0595	0.5338	0.1426	0.2918	0.4174	0.2263	0.4232	0.1047
FOR × TA			0.0039	0.9204	0.2572	0.9477	0.1581	0.0032	0.3946	0.085	0.1036
FOR × AD			0.4487	0.1418	0.3068	0.0707	0.0207	0.4497	0.1478	0.0126	0.5671
TA × AD			0.1458	0.3725	0.4238	0.4177	0.6657	0.1585	0.4195	0.7165	0.2384
FOR × TA × AD			0.0429	0.2565	0.2986	0.0611	0.5454	0.044	0.0214	0.0493	0.0086

^1^*b* = asymptotic CH_4_ production (mL/g DM); *c* = rate of CH_4_ production (mL/h); *Lag* = initial delay before CH_4_ production begins (h)

^2^SEM = standard error of mean

FOR, Forages; TA, Types of additives; AD, Additive doses

Mitigation in asymptotic CH_4_ production was obtained by adding the extract (36.83 mL/g DM) and chitosan nanoparticles into the corn silage forage. But the supplementation of chitosan into this forage increased asymptotic CH_4_ production with respect to the control. Likewise, the incorporation of extract and chitosan nanoparticles reduced the rate of CH_4_ production as compared to the control. However, the lag period was increased due to the supplementation of chitosan only. The inclusion of extract and chitosan nanoparticles at 1 mL/g DM depicted CH_4_ mitigation from 50.08 mL gas/g DM incubated (control) to 36.79 and 34.81 mL gas/g DM incubated, respectively. In addition to this, all additives showed significantly (*P* < 0.0001) lower CH_4_ production at 48 h than the control.

Asymptotic CH_4_ production was reduced when oats hay forage was supplemented with the extract (40.63 mL/g DM) and chitosan nanoparticles (47.77 mL/g DM). The rate of CH_4_ production was more or less unaffected (*P* = 0.0414) due to the inclusion of additives. On the other hand, the incorporation of additives into the diet significantly (*P* = 0.0002) increased the lag period. As compared to the control and other additives (chitosan and chitosan nanoparticles), the extract mitigated CH_4_ production (40.63 mL gas/g DM incubated and 9.84 ml CH_4_/100 mL gas) at 48 h.

###  CO gas production

Table 5 illustrates the effect of different additives at varied doses on ruminal CO production from bulls using alfalfa hay, corn silage, and oats hay as forages. The supplementation of extract (0.25 mL/g DM) in alfalfa hay showed a reduction in asymptotic CO gas production (0.1149 mL/g DM) when compared to the control (0.2454 mL/g DM). Asymptotic CO gas production was increased due to the inclusion of chitosan nanoparticles at different concentrations. The rate of CO gas production was not affected (*P* = 0.7991) after the supplementation of additives. Likewise, all the additives increased the lag period, but the effect was not significant (*P* = 0.7081). As compared to the control (0.1216 mL/g DM incubated) and additives (chitosan—0.1025 mL/g DM incubated; chitosan nanoparticles—0.1394 mL/g DM incubated), the extract significantly (*P* < 0.0001) reduced CO production (0.0582 mL/g DM incubated) at 48 h.

**Table 5:** Effect of different additives at varied doses (0.25, 0.5, and 1 mL/g dietary DM) on ruminal CO production from bulls using alfalfa hay, corn silage, and oats hay as forages

Forages (FOR)	Types of additives (TA)	Additive doses (AD; mL/g DM)	CO production kinetics^1^			CO production (mL/g DM incubated)		
			*b*	*c*	*Lag*	6 h	24 h	48 h
Alfalfa hay	No additive	0	0.2454	0.0010	0.2107	0.0164	0.0386	0.1216
	Chitosan	0.25	0.2056	0.0013	0.2365	0.0016	0.0125	0.1025
		0.5	0.2071	0.0013	0.2388	0.0020	0.0131	0.1031
		1	0.2827	0.0013	0.2424	0.0014	0.0153	0.1412
	Crude extract	0.25	0.1149	0.0012	0.2343	0.0021	0.0105	0.0582
		0.5	0.1179	0.0012	0.2400	0.0019	0.0078	0.0589
		1	0.1359	0.0012	0.2414	0.0023	0.0083	0.0679
	Chitosan nanoparticles	0.25	0.2809	0.0009	0.2100	0.0182	0.0436	0.1394
		0.5	0.3961	0.0034	0.2068	0.0392	0.0645	0.1966
		1	0.3626	0.0005	0.2387	0.0313	0.0527	0.1619
	TA		< 0.0001	0.7991	0.7081	< 0.0001	< 0.0001	< 0.0001
	AD		0.1017	0.2915	0.8649	0.034	0.3632	0.2027
	TA × AD		0.3098	0.2702	0.9912	0.0175	0.2315	0.2184
Corn silage	No additive	0	0.2380	0.0007	0.1881	0.0039	0.0054	0.0303
	Chitosan	0.25	0.1472	0.0016	0.2400	0.0005	0.0068	0.0841
		0.5	0.1287	0.0015	0.2422	0.0010	0.0066	0.0759
		1	0.1712	0.0012	0.2458	0.0009	0.0059	0.0704
	Crude extract	0.25	0.1053	0.0010	0.2374	0.0020	0.0068	0.0526
		0.5	0.1225	0.0009	0.2418	0.0020	0.0068	0.0577
		1	0.0964	0.0010	0.2339	0.0025	0.0073	0.0430
	Chitosan nanoparticles	0.25	0.0895	0.0006	0.1899	0.0032	0.0057	0.0405
		0.5	0.0701	0.0009	0.2209	0.0035	0.0074	0.0421
		1	0.0676	0.0003	0.1751	0.0028	0.0052	0.0338
	TA		< 0.0001	0.0026	0.9506	< 0.0001	0.0312	< 0.0001
	AD		0.717	0.3341	0.9986	0.4559	0.148	0.0521
	TA × AD		0.9277	0.184	1	0.304	0.6291	0.524
Oats hay	No additive	0	0.0749	0.0005	0.4431	0.0050	0.0093	0.0370
	Chitosan	0.25	0.1889	0.0018	0.2411	0.0004	0.0074	0.1105
		0.5	0.1948	0.0014	0.2411	0.0005	0.0081	0.1026
		1	0.1981	0.0011	0.2434	0.0005	0.0068	0.0826
	Crude extract	0.25	0.1087	0.0009	0.2427	0.0014	0.0057	0.0538
		0.5	0.1288	0.0010	0.2446	0.0011	0.0058	0.0592
		1	0.1016	0.0011	0.2431	0.0015	0.0052	0.0465
	Chitosan nanoparticles	0.25	0.0917	0.0008	0.2326	0.0057	0.0091	0.0420
		0.5	0.0919	0.0011	0.2212	0.0056	0.0099	0.0503
		1	0.0777	0.0007	0.2236	0.0033	0.0060	0.0390
	TA		0.3245	0.0003	0.0093	< 0.0001	0.4245	< 0.0001
	AD		0.9892	0.1631	0.5255	0.7243	0.484	0.1958
	TA × AD		0.9783	0.4424	0.7332	0.5815	0.4781	0.905
SEM pooled^2^			0.02490	0.00020	0.01685	0.00079	0.00171	0.00885
*P* value:								
FOR			< 0.0001	0.2719	0.0211	< 0.0001	< 0.0001	< 0.0001
TA			0.0005	0.2106	0.3944	< 0.0001	< 0.0001	< 0.0001
AD			0.6146	0.1017	0.9868	0.0268	0.2333	0.3423
FOR × TA			< 0.0001	0.3414	0.9817	< 0.0001	< 0.0001	< 0.0001
FOR × AD			0.6174	0.5114	0.9885	0.0083	0.4595	0.0577
TA × AD			0.7331	0.0786	1	0.011	0.1394	0.281
FOR × TA × AD			0.9567	0.4615	0.9999	0.0013	0.2561	0.2717

^1^*b* = asymptotic CO production (mL/g DM); *c* = rate of CO production (mL/h); *Lag* = initial delay before CO production begins (h)

^2^ SEM = standard error of mean

FOR, forages; TA, types of additives; AD, additive doses

In the presence of corn silage as forage, the supplementation of chitosan, extract, and chitosan nanoparticles in the diet reduced the asymptotic CO production significantly (*P* < 0.0001). However, the rate of CO production was decreased only due to the inclusion of chitosan nanoparticles. On the other hand, all the additives increased the lag period with respect to the control, but the effect was not significant (*P* = 0.9506). Surprisingly, the incorporation of chitosan, extract, and chitosan nanoparticles in the diet enhanced CO production at 48 h.

The addition of chitosan, extract, and chitosan nanoparticles in the diet containing oats hay forage increased asymptotic CO production (*P* = 0.3245) and rate of CO production (*P* = 0.0003) but decreased the lag period (*P* = 0.0093) as compared to the control. On the other hand, chitosan, extract, and chitosan nanoparticles-supplemented forage increased CO production significantly (*P* < 0.0001) at 48 h with respect to the control.

###  H_2_S production

Table 6 shows the effect of different additives on ruminal H_2_S production from bulls using alfalfa hay, corn silage, and oats hay as forages. In the presence of alfalfa hay as forage, the supplementation of additives reduced asymptotic H_2_S production significantly (*P* < 0.0001). Similarly, among varied additives used, only the inclusion of chitosan nanoparticles showed reduction in the rate of H_2_S gas production as compared to the control. On the other hand, all additives increased the lag period, but the effect was not significant (*P* = 0.7478). The addition of chitosan at 1 mL/g DM depicted maximum mitigation of H_2_S production (0.0740 mL/g DM incubated) with respect to the control (0.1695 mL/g DM incubated).

**Table 6:** Effect of different additives at varied doses (0.25, 0.5, and 1 mL/g dietary DM) on ruminal H_2_S production from bulls using alfalfa hay, corn silage, and oats hay as forages

Forages (FOR)	Types of additives (TA)	Additive doses (AD; mL/g DM)	H_2_S production kinetics^1^			H_2_S production (mL/g DM incubated)		
			*b*	*c*	*Lag*	6 h	24 h	48 h
Alfalfa hay	No additive	0	0.3392	0.0009	0.2164	0.0206	0.0386	0.1695
	Chitosan	0.25	0.1486	0.0014	0.2445	0.0004	0.0057	0.0744
		0.5	0.1533	0.0012	0.2382	0.0006	0.0075	0.0766
		1	0.1489	0.0013	0.2386	0.0006	0.0100	0.0740
	Crude extract	0.25	0.2922	0.0012	0.2382	0.0009	0.0188	0.1458
		0.5	0.3183	0.0011	0.2312	0.0051	0.0275	0.1585
		1	0.3476	0.0012	0.2354	0.0033	0.0252	0.1731
	Chitosan nanoparticles	0.25	0.2576	0.0009	0.2104	0.0141	0.0362	0.1281
		0.5	0.3012	0.0007	0.1706	0.0209	0.0424	0.1472
		1	0.3414	0.0007	0.2895	0.0185	0.0442	0.1702
	TA		< 0.0001	< 0.0001	0.7478	< 0.0001	< 0.0001	< 0.0001
	AD		0.3619	0.3224	0.2106	0.1528	0.1404	0.385
	TA × AD		0.8751	0.9563	0.2169	0.7004	0.9192	0.876
Corn silage	No additive	0	0.3276	0.0007	0.2209	0.0181	0.0368	0.1408
	Chitosan	0.25	0.2182	0.0016	0.2437	0.0002	0.0079	0.1214
		0.5	0.2310	0.0013	0.2428	0.0007	0.0099	0.1147
		1	0.2459	0.0012	0.2455	0.0004	0.0097	0.1058
	Crude extract	0.25	0.3790	0.0016	0.2337	0.0134	0.0352	0.2307
		0.5	0.4386	0.0011	0.2406	0.0092	0.0277	0.1797
		1	0.4067	0.0011	0.2261	0.0129	0.0399	0.2077
	Chitosan nanoparticles	0.25	0.3206	0.0009	0.2251	0.0086	0.0261	0.1556
		0.5	0.3039	0.0008	0.2187	0.0096	0.0319	0.1538
		1	0.1725	0.0005	0.2231	0.0045	0.0137	0.0836
	TA		0.139	0.0002	< 0.0001	< 0.0001	< 0.0001	0.0118
	AD		0.9472	0.9347	0.5424	0.0286	0.5821	0.3877
	TA × AD		0.0989	0.4649	0.0027	0.0002	0.0001	0.0036
Oats hay	No additive	0	0.3930	0.0008	0.2162	0.0214	0.0434	0.1958
	Chitosan	0.25	0.2854	0.0012	0.2533	0.0002	0.0066	0.1191
		0.5	0.2473	0.0014	0.2470	0.0002	0.0087	0.1404
		1	0.2604	0.0012	0.2475	0.0001	0.0059	0.1194
	Crude extract	0.25	0.2479	0.0013	0.2347	0.0075	0.0197	0.1379
		0.5	0.3101	0.0011	0.2408	0.0017	0.0142	0.1442
		1	0.3529	0.0011	0.2289	0.0069	0.0333	0.1872
	Chitosan nanoparticles	0.25	0.3472	0.0008	0.2174	0.0144	0.0353	0.1725
		0.5	0.3472	0.0007	0.2092	0.0178	0.0430	0.1812
		1	0.2680	0.0008	0.2288	0.0040	0.0177	0.1184
	TA		0.0002	0.0011	0.0002	0.0008	0.0003	0.0001
	AD		0.4139	0.0219	0.7825	0.8488	0.8998	0.1465
	TA × AD		0.2622	0.7819	0.3603	0.6848	0.2136	0.2612
SEM pooled^2^			0.03387	0.00012	0.00706	0.00149	0.00357	0.01642
*P* value:								
FOR			0.0653	0.9665	0.9873	0.6576	0.6378	0.0722
TA			< 0.0001	< 0.0001	0.0144	< 0.0001	< 0.0001	< 0.0001
AD			0.6443	0.018	0.2077	0.3628	0.564	0.7486
FOR × TA			0.001	0.9441	0.9637	< 0.0001	< 0.0001	0.001
FOR × AD			0.4614	0.1446	0.1787	0.203	0.5643	0.0885
TA × AD			0.1543	0.7836	0.0469	0.0206	0.0017	0.0553
FOR × TA × AD			0.4233	0.8596	0.334	0.2438	0.073	0.2802

^1^*b* = is the asymptotic H_2_S production (mL/g DM); *c* = is the rate of H_2_S production (mL/h); *Lag* = is the initial delay before H_2_S production begins (h)

^2^ SEM = standard error of mean

FOR, forages; TA, types of additives; AD, additive doses

Using corn silage as substrate, the supplementation of chitosan (0.2182 mL/g DM) and chitosan nanoparticles (0.1725 mL/g DM) mitigated the release of asymptotic H_2_S production, but the extract caused increment in asymptotic H_2_S production (0.4386 mL/g DM) as compared to the control (0.3276 mL/g DM). All the additives significantly (*P* = 0.0002) increased the rate of H_2_S production, but the supplementation of chitosan nanoparticles (1 mL/g DM) slightly reduced the rate of gas production (0.0005 mL/h) with respect to the control (0.0007 mL/h). In a like manner, the incorporation of all additives increased the lag period as compared to the control. The supplementation of chitosan nanoparticles (1 mL/g DM) demonstrated maximum mitigation of H_2_S production (0.0836 mL/g DM incubated) with respect to the control (0.1408 mL/g DM incubated) at 48 h. However, H_2_S gas production increased due to the addition of extract at all concentrations.

The supplementation of chitosan, extract, and chitosan nanoparticles at different concentrations in oats hay forage significantly (*P* = 0.0002) reduced asymptotic H_2_S gas production with respect to the control. On the other hand, only chitosan nanoparticles (0.5 mL/g DM; 0.0007 mL/h) as an additive showed a slight reduction in the rate of H_2_S gas production as compared to the control (0.0008 mL/h). The inclusion of all additives increased the lag period significantly (*P* = 0.0002). Interestingly, the addition of chitosan, extract, and chitosan nanoparticles at different concentrations significantly (*P* = 0.0001) mitigated H_2_S gas production (mL/g DM incubated) at 48 h.

### Fermentation profile and CH_4_ conversion efficiency

The effect of different additives on rumen fermentation profile and CH_4_ conversion efficiency from bulls using alfalfa hay, corn silage, and oats hay as forages is summarized in Table 7. The addition of chitosan (0.25 mL/g DM) in alfalfa hay increased the pH and DMD to 7.3 and 78.95%, respectively, while the pH and DMD were more or less similar to the control (pH—6.6; DMD—67.58%) after the addition of extract and chitosan nanoparticles. On the other hand, the inclusion of chitosan at varied concentrations decreased the SCFA, while SCFA was increased due to the supplementation of extract and chitosan nanoparticles. Likewise, the addition of chitosan showed a reduction in ME, while an increment in ME was observed with respect to the control due to the inclusion of extract and chitosan nanoparticles. All the additives significantly affected CH_4_:ME (*P* = 0.0002), CH_4_:OM (*P* = 0.0001), and CH_4_:SCFA (*P* < 0.0001).

**Table 7:** Effect of different additives at varied doses (0.25, 0.5, and 1 mL/g dietary DM) on rumen fermentation profile and CH_4_ conversion efficiency from bulls using alfalfa hay, corn silage, and oats hay as forages

Forages (FOR)	Types of additives (TA)	Additive doses (AD; mL/g DM)	Rumen fermentation profile			CH_4_ conversion efficiency			
			pH	DMD (%)	SCFA (mmol/g DM)	ME (MJ/kg DM; 24 h)	CH_4_:ME (g/MJ)	CH_4_:OM (ml/g)	CH_4_:SCFA (mmol/mmol; 24 h)
Alfalfa hay	No additive	0	6.6	67.58	6.85	6.82	5.99	9.89	37.09
	Chitosan	0.25	7.3	78.95	4.36	5.54	4.70	6.33	37.16
		0.5	7.2	72.07	4.78	5.75	3.76	5.24	28.18
		1	7.2	71.73	4.84	5.79	2.90	4.06	21.63
	Crude extract	0.25	6.9	67.94	7.74	7.27	3.37	5.90	19.85
		0.5	6.6	65.51	9.31	8.08	4.20	8.22	22.67
		1	6.2	67.99	10.74	8.81	3.62	7.74	18.52
	Chitosan nanoparticles	0.25	6.6	64.91	7.22	7.01	4.92	8.31	29.88
		0.5	6.5	66.27	6.71	6.74	6.06	9.89	37.96
		1	6.2	63.18	7.17	6.98	6.25	10.57	37.88
	TA		< 0.0001	0.03	< 0.0001	< 0.0001	0.0002	0.0001	< 0.0001
	AD		< 0.0001	0.6387	0.0041	0.0041	0.6209	0.5071	0.3678
	TA × AD		< 0.0001	0.823	0.0048	0.0048	0.103	0.171	0.0225
Corn silage	No additive	0	6.7	61.10	7.64	7.22	6.44	11.29	37.80
	Chitosan	0.25	7.4	69.98	5.04	5.89	3.65	5.19	26.65
		0.5	7.4	67.55	5.44	6.09	5.19	7.64	36.24
		1	7.3	72.71	4.99	5.86	4.07	5.77	29.70
	Crude extract	0.25	6.4	48.05	10.42	8.65	2.79	5.85	14.38
		0.5	6.6	54.65	10.80	8.85	3.62	7.69	18.52
		1	6.2	46.26	10.24	8.56	4.09	8.51	21.36
	Chitosan nanoparticles	0.25	6.6	60.19	7.44	7.12	5.66	9.71	33.81
		0.5	6.6	62.35	7.88	7.35	5.17	9.17	30.02
		1	6.6	59.97	7.53	7.17	5.75	9.95	34.10
	TA		< 0.0001	< 0.0001	< 0.0001	< 0.0001	< 0.0001	< 0.0001	< 0.0001
	AD		0.8856	0.0009	0.0011	0.0011	0.0243	0.0183	0.021
	TA × AD		0.1481	0.0103	0.0002	0.0002	0.6084	0.7932	0.3839
Oats hay	No additive	0	5.8	58.19	6.75	6.76	7.81	12.74	48.87
	Chitosan	0.25	7.1	72.33	4.35	5.53	3.81	5.09	30.38
		0.5	7.2	66.80	4.98	5.86	2.59	3.66	19.00
		1	7.3	59.15	4.90	5.81	4.84	6.81	35.82
	Crude extract	0.25	6.0	38.85	9.57	8.21	4.39	8.71	23.54
		0.5	5.9	54.77	7.37	7.08	3.30	5.68	19.63
		1	5.9	36.02	10.07	8.47	4.09	8.43	21.36
	Chitosan nanoparticles	0.25	5.8	56.90	6.98	6.88	8.43	14.02	51.77
		0.5	5.7	59.02	7.09	6.94	5.59	9.37	34.18
		1	5.7	54.51	7.56	7.18	7.43	12.90	43.98
	TA		< 0.0001	< 0.0001	< 0.0001	< 0.0001	0.0072	0.0156	0.0004
	AD		0.0001	0.0877	0.2803	0.2798	0.4748	0.4488	0.5042
	TA × AD		0.0017	0.0004	0.9974	0.9974	0.5361	0.6492	0.465
SEM pooled^3^			0.03	2.235	0.269	0.138	0.535	0.992	3.146
*P* value:									
FOR			< 0.0001	< 0.0001	< 0.0001	< 0.0001	0.0389	0.0865	0.0132
TA			< 0.0001	< 0.0001	< 0.0001	< 0.0001	< 0.0001	< 0.0001	< 0.0001
AD			< 0.0001	0.0136	0.0037	0.0037	0.47	0.2917	0.422
FOR × TA			< 0.0001	< 0.0001	0.0507	0.0508	0.0924	0.1581	0.0852
FOR × AD			< 0.0001	0.0306	0.0011	0.0011	0.0206	0.0207	0.0126
TA × AD			< 0.0001	0.0235	0.0129	0.0129	0.7494	0.6658	0.7157
FOR × TA × AD			0.0001	0.1827	0.0003	0.0003	0.2385	0.5455	0.0486

^1^pH, ruminal pH; DMD, dry matter degradability; SCFA, short-chain fatty acids; ME, the metabolizable energy

^2^CH_4_:SCFA = methane:short-chain fatty acids ratio; CH_4_:ME = methane:metabolizable energy ratio; CH_4_:OM = methane:organic matter ratio

^3^SEM, standard error of the mean; FOR, Forages; TA, Types of additives; AD, Additive doses

Using corn silage as forage, pH was increased due to the supplementation of chitosan, while pH level was reported more or less similar to the control in the presence of extract and chitosan nanoparticles. Similarly, an increase in DMD (72.71%) was estimated with respect to the control (61.1%) in the presence of chitosan, while DMD was reduced due to the addition of extract and chitosan nanoparticles. The inclusion of chitosan revealed a reduction in SCFA, while the extract and chitosan nanoparticles increased the SCFA as compared to the control. The ME, CH_4_:ME, CH_4_:OM, and CH_4_:SCFA were significantly (*P* < 0.0001) affected due to the incorporation of all the additives.

Ruminal pH was increased from 5.8 (control) to 7.3 because of the addition of chitosan in the oats hay forage. On the other hand, pH was calculated more or less similar to the control by supplementing the extract and chitosan nanoparticles. In a like manner, DMD was improved due to the inclusion of chitosan at different concentrations. The addition of chitosan decreased SCFA level, while the extract and chitosan nanoparticles improved SCFA level with respect to the control. The supplementation of chitosan, extract, and chitosan nanoparticles significantly (*P* < 0.05) affected ME, CH_4_:ME, CH_4_:OM, and CH_4_:SCFA.

## Discussion

The consumption of cellulase-based non-human edible food resources by ruminants leads to the emission of GHG, particularly CH_4_ via exhalation and eructation process (McAllister and Newbold 2008). In general, methanogens utilize H_2_ to reduce CO_2_ into CH_4_ during anaerobic fermentation of carbohydrates present in the rumen (Jafari et al. 2019). The global warming caused due to the release of CH_4_ into the atmosphere is 27 times higher than that of CO_2_. Of total anthropogenic gases emitted by livestock, approximately 3.5% is associated with the production of CH_4_, leading to extensive energy loss in ruminants (Rey et al. 2023). Therefore, potential feed additives are needed of this hour which should not only be non-toxic in nature and maintain the animals’ productivity but also be economically viable towards the mitigation of CH_4_ emission.

In the present study, *Y. schidigera* extract, chitosan, and chitosan nanoparticles were observed as potent additives in terms of quantifying GHG production from bulls by supplementing them in alfalfa hay, corn silage, and oats hay forages. *Y. schidigera* extract enhanced gas production kinetics and total gas production from bulls using alfalfa hay, corn silage, and oats hay forages. A similar observation was reported by Lila et al. (2003) and Singer et al. (2008) who estimated increased total gas production from ruminants in the presence of *Y. schidigera* extract. In contrary, Zeid et al. (2019) estimated a reduction in total gas production from sheep when the feeding diet was supplemented with *Y. schidigera* extract. In a like manner, Soliman (2022) estimated the total gas production-reducing ability of *Y. schidigera* extract as an additive in cow’s feed. In a different study, Xu et al. (2010) demonstrated that the supplementation of *Y. schidigera* extract in different types of forages showed no effect on total gas production, rate of gas production, and lag period from steers. On the other hand, despite very limited investigations on deciphering the role of chitosan towards total gas production from ruminants, Wencelová et al. (2014) and Jiménez-Ocampo et al. (2021) depicted no effect of chitosan on total gas production from sheep and crossbred heifers, respectively. To the best of our knowledge, no prior reports investigated the effect of chitosan nanoparticles on total gas production from ruminants, hence, comparative analyses are not discussed here. However, in this context, the supplementation of chitosan and chitosan nanoparticles in alfalfa hay, corn silage, and oats hay forages reduced the gas production kinetics and total gas production from bulls as compared to *Y. schidigera* extract.

Ruminants release an extensive amount of CH_4_ into the environment via the fermentation of disparate feed consumed (Pedraza-Hernández et al. 2019; Anele et al. 2022). The emission of enteric CH_4_ from ruminants relies on several factors, such as the physico-chemical properties of rations, composition of rations, fermentation pattern, and passage rate of digests from the rumen (Broucek 2018). The fermentation of cellulose and hemicellulose present in the feeds causes increased acetate:propionate production and higher emission of CH_4_ (Ellis et al. 2012). Over the past few decades, several strategies viz. utilization of probiotics and prebiotics, vaccine administration, defaunation, and inclusion of natural bioactive compounds in feed have been implemented by worldwide researchers to mitigate CH_4_ emission from livestock (Jiménez-Ocampo et al. 2019; Velázquez et al. 2020). However, its practical applicability and certain variabilities are the major hurdles for veterinarians. In recent years, the direct manipulation of feeding diets by adding supplements is showcasing enormous significance in terms of reducing the level of CH_4_ emission from animals, without altering the ruminal fermentation mechanism (Khusro et al. 2022). These dietary supplements are known to mitigate CH_4_ production by targeting methanogenesis, inhibiting the growth of protozoa, and inducing propionate production (Jiménez-Ocampo et al. 2019). In the present study, CH_4_ production was reduced from bulls by supplementing either of the additives (*Y. schidigera* extract or chitosan or chitosan nanoparticles) in the feed but the effect was forage dependent. In line of our study, Xu et al. (2010) and Anele et al. (2022) also reported a reduction in CH_4_ emission due to the inclusion of *Y. schidigera* extract in the ruminants’ diet. In contrary, our findings were inconsistent with the report of Zijderveld et al. (2011) who demonstrated no reduction in the production of CH_4_ from the lactating cows due to the supplementation of *Y. schidigera* powder. Similarly, our findings disagree with the reports of Henry et al. (2015), Jiménez-Ocampo et al. (2021), and Rey et al. (2023) who demonstrated no influence of chitosan as an additive on enteric CH_4_ emission from ruminants. To the best of our knowledge, previously, no studies reported the CH_4_ mitigating effect of chitosan nanoparticles from ruminants, hence, comparative findings are not discussed here.

Carbon monoxide is a weak GHG but shows significant indirect effects on the environment (Santillán et al. 2023). While it does not directly trap heat like CO_2_ or CH_4_, CO increases the concentration and lifespan of more potent GHG, particularly CH_4_, by reacting with hydroxyl radicals that would otherwise break down CH_4_. Additionally, CO affects the transfer of gases between the troposphere and stratosphere, influencing the stability of the ozone layer. Thus, CO indirectly contributes to global warming and ozone depletion, making its environmental impact more substantial than it initially appears (Sobieraj et al. 2020). In this investigation, the decrement or increment in CO production from bulls using additives (*Y. schidigera* extract, chitosan, and chitosan nanoparticles) was observed to be a forage-dependent mechanism. Previously, Santillán et al. (2023) depicted a reduction in the production of CO from horse in the presence of plant leaf extract as an additive. However, recently, Elghandour et al. (2024) observed a significant enhancement in CO production from steers using *M. oleifera* seeds as dietary supplements. To the best of our knowledge, this is the first report on deciphering the effect of *Y. schidigera* extract, chitosan, and chitosan nanoparticles on CO production from bulls, therefore, no direct comparative studies are discussed here.

Hydrogen sulphide is recognized as a toxic signaling molecule, alongside nitric oxide and CO (Shah et al. 2020). The anaerobic digestion of organic materials by sulfate-reducing bacteria releases H_2_S into the ecosystem. In animals, gut bacteria metabolize dietary sulfate, producing H_2_S, which is rapidly absorbed through the intestinal wall, leading to toxic effects (Pal et al. 2018). Accumulation of H_2_S gas can cause conditions like poliomyelitis in ruminants, making it crucial to regulate H_2_S synthesis in the rumen (Binversie et al. 2016). Sulfide production in the rumen is influenced by dietary sulfate levels, as ruminal microbes utilize sulfur or sulfates to synthesize sulfides, increasing H_2_S concentration. Methanogens and sulfate-reducing bacteria exhibit a competitive relationship, as both require hydrogen for their metabolic processes. Sulfate-reducing bacteria reduce sulfate to sulfide, while methanogens reduce CO_2_ to CH_4_ in the rumen (Shah et al. 2020). Recent findings of Santillán et al. (2023) and Elghandour et al. (2024) confirmed H_2_S mitigating traits of plants as additives from horses and steers, respectively. In the current context, the inclusion of additives mitigated H_2_S production from bulls, but the effect was observed to be a forage-dependent process, thereby favouring the findings of prior reports. Anele et al. (2022) observed no significant effect of *Y. schidigera* supplementation on H_2_S gas production from dairy cows. In contrast to this, feeding a sulfur-containing diet to steers increased the rate of H_2_S production. Since, there is a dearth of reports exploring the role of chitosan and chitosan nanoparticles in the mitigation of H_2_S production from animals, hence, direct comparison is not discussed here.

Plants-associated secondary metabolites are potent antimicrobial and antiprotozoal agents that help in controlling ruminal microflora. These metabolites show concentration-dependent growth inhibitory and inducing activities against ruminal microbes (Anele et al. 2022). Saponin is the main bioactive component of *Y. schidigera* and it is present in steroidal form. In general, saponin reduces GHG by exhibiting antiprotozoal activity. Thus, the emission of GHG from livestock can be controlled by decreasing the total count of protozoa (Adegbeye et al. 2019). Saponins attach to the sterols present in the membrane of protozoa and cause cell death by lysing the cell membrane. It also selectively inhibits certain groups of ruminal bacteria. Thus, *Y. schidigera*-associated saponins are considered to improve feed utilization efficiency and inhibit the mechanism of GHG emission, particularly methanogenesis for CH_4_ production (Zeid et al. 2019). Additionally, *Y. schidigera* reduces CH_4_ emission by inhibiting the growth of cellulase-producing microbes (Adegbeye et al. 2019).

Similarly, chitosan as feed additive affects the fermentation and enteric GHG production from ruminants. It is speculated that the inhibition of GHG emission pathway or methanogens is mainly due to the electrostatic interaction of chitosan and the destabilization of the microbial cell membrane (Jiménez-Ocampo et al. 2019). In addition, the inclusion of chitosan as an additive alters the molar proportions of volatile fatty acids in the rumen which leads to the enhanced utilization of metabolizable energy for growth, followed by a decrease in GHG production (Jiménez-Ocampo et al. 2019).

In this study, the supplementation of *Y. schidigera* extract, chitosan, and chitosan nanoparticles as additives increased pH and DMD levels. The CH_4_:ME, CH_4_:OM, and CH_4_:SCFA were significantly (*P* < 0.05) affected due to the inclusion of these additives in the forages. Findings more or less similar to our results were reported by Xu et al. (2010), Zeid et al. (2019), Soliman (2022), and Anele et al. (2022) who used *Y. schidigera* as additives in the feeding diet of ruminants.

In conclusion, the supplementation of *Y. schidigera* extract, chitosan, and chitosan nanoparticles in alfalfa hay, corn silage, and oats hay forage increased total gas production from bulls. Interestingly, mitigation in CH_4_, CO, and H_2_S productions were observed by supplementing either of the additives (*Y. schidigera* extract or chitosan or chitosan nanoparticles) in the forage but the effect was dependent on the type of forage used. Additionally, the fermentation profiles, such as pH and DMD were increased, while CH_4_ conversion efficiency viz. CH_4_:ME, CH_4_:OM, and CH_4_:SCFA were significantly (*P* < 0.05) affected due to the incorporation of respective additives in the forages. Thus, this in vitro investigation established the fundamental aspects of exploiting *Y. schidigera* extract, chitosan, and chitosan nanoparticles as prominent feed additives to mitigate the emission of GHG from bulls towards sustainable and cleaner ecosystem.

## Data Availability

The datasets used and/or analysed during the current study are available from the corresponding author on reasonable request.
